# Integrating Bacterial Identification and Susceptibility Testing: A Simple and Rapid Approach to Reduce the Turnaround Time in the Management of Blood Cultures

**DOI:** 10.1155/2019/8041746

**Published:** 2019-10-03

**Authors:** Dariane C. Pereira, Luciano Z. Goldani

**Affiliations:** ^1^Microbiology Unit, Laboratory Diagnosis Service, Hospital de Clínicas de Porto Alegre, Universidade Federal do Rio Grande do Sul, Porto Alegre, Brazil; ^2^InfectiousDiseases Unit, Hospital de Clínicas de Porto Alegre, Universidade Federal do Rio Grande do Sul, Porto Alegre, Brazil

## Abstract

We evaluated a rapid bacterial identification (rID) and a rapid antimicrobial susceptibility testing by disk diffusion (rAST) from positive blood culture to overcome the limitations of the conventional methods and reduce the turnaround time in bloodstream infection diagnostics. The study included hemocultures flagged as positive by bacT/ALERT®, identification by MALDI-TOF MS, and rAST. The results were compared to identification and antimicrobial susceptibility testing (AST) results by current standard methods, after 24 h incubation. For rAST categorical agreement (CA), very major errors (VME), major errors (ME), and minor errors (mE) were calculated. A total of 524 bacterial samples isolated from blood cultures were obtained, including 246 Gram-negative (GN) and 278 Gram-positive (GP) aerobes. The overall concordance of rID was 88.6%, and it was highest among GN (96%). A total of 2196 and 1476 antimicrobial agent comparisons were obtained for GN and GP, respectively. Evaluation of rAST, CA, VME, ME, and mE disclosed 97.7, 0.7, 0.5, and 1.1% for GN and 98.0, 0.5, 0.7, and 0.8% for GP, respectively. Meropenem CA, VME, and ME were 98.3, 0.5, and 0.5%, respectively; mE was not observed. Oxacillin CA, ME, and mE were 97.4, 1.6, and 0.6%, respectively; VME was not observed. Overall, kappa scores of the results of the comparisons demonstrated the high agreement between rAST and the standard method. Identification and AST of aerobic bacteria from positive blood cultures after a short period of incubation on solid blood agar is a fast and reliable method that may improve the management of bloodstream infections.

## 1. Introduction

The development of rapid diagnostic assays for the identification and analysis of antimicrobial resistance of bacteria causing bloodstream infections is of utmost importance to reduce morbidity and mortality. Infectious diseases have a substantial global health impact. Fast diagnosis of pathogens is critical to guarantee adequate therapy for infections [1]. A wide variety of microorganisms can cause bloodstream infections, commonly *E. coli*, *Klebsiella* spp., *S. aureus*, other bacteria, and yeast. Rapid identification of bloodstream pathogens is a laboratory practice that supports fast transitions to direct target therapy, supporting timely and effective patient care [2]. Current technologies employed in routine diagnostics are based on bacterial culture, which constitutes the actual gold standard and are precise and sensitive but rather slow. Traditional identification and antimicrobial susceptibility test results for microorganisms causing bloodstream infections can take 48 h or longer to obtain. Today, new methods have been made available to enable faster diagnosis. Recently, matrix-assisted laser desorption/ionization time-of-flight mass spectrometry (MALDI-TOF MS) emerged as a rapid, accurate, cost-effective method and has been effectively used as a fast method for identifying a wide array of microbial species [3]. In MALDI-TOF MS analysis, abundant structural proteins such as ribosomal proteins are extracted from an entire bacterial colony. The ionizing laser vaporizes structural proteins of microorganisms, and unique mass spectra are generated, having mass-to-charge ratio (*m*/*z*) peaks with varying intensities. The mass spectra of test isolates are sequentially compared with those in a reference database for identification [4].

In comparison with conventional methods for identification of clinical samples by and despite its high-end technology, a MALDI-TOF MS device is simple to use. MALDI-TOF MS can provide advantages for a universal procedure of microbial identification. Only a small amount of an organism, typically a fraction of a single colony from primary culture plates, is required for analysis. Comparatively, a larger inoculum and subculture is often required for conventional biochemical methods or other automated systems. Furthermore, once the instrument is loaded, identifications can typically be performed in less than one minute, compared with hours to days for conventional methods [4, 5].

Even simpler and faster than traditional bacterial identification methods, analysis of blood cultures via MALDI-TOF MS and the detection of antimicrobial resistance require a preliminary bacterial culture in solid media. In this study, we evaluated a rapid modified bacterial identification and an early antimicrobial resistance detection from positive blood cultures based on centrifugation and short-time bacterial incubation to reduce the turnaround time in bloodstream infection diagnostics in a routine microbiology laboratory.

## 2. Materials and Methods

### 2.1. Study Design and Study Population

Transversal study: all Gram-negative bacilli and *Staphylococcus* sp. isolates recovered from bloodstream infection during 2017 and 2018 of patients admitted at a tertiary hospital in southern of Brazil were included in the study.

### 2.2. Blood Culture Procedures

Blood culture sets were obtained from patients with bloodstream infection. Aerobic and anaerobic blood culture bottles (bacT/ALERT® culture media FA Plus, PF plus and FN Plus, bioMérieux, France) were forwarded to the Microbiology Unit and incubated in the automated blood culture system bacT/ALERT® 3D (bioMérieux). Negative bottles are automatically resulted and discarded after five days of incubation. The performance methods were evaluated for positive blood cultures with monomicrobial bacterial growth. Bottles yielding polymicrobial or yeast growth were excluded. For analyses, we considered only one blood culture series per individual patient. Moreover, the testing only included blood cultures from the daily microbiology laboratory routine; no artificially inoculated vials were evaluated in this study.

### 2.3. Standard Bacterial Identification (sID) and Antimicrobial Susceptibility Testing (sAST) from Positive Blood Culture

Blood culture flasks were analyzed after bacT/ALERT®3D system flagged a blood as positive. The blood culture flasks were analyzed by Gram staining followed by subculture on an appropriate solid agar medium (aerobic Columbia agar 5% sheep blood, chocolate agar, MacConkey agar, bioMérieux, France) following 18–24 h incubation at 35°C and 5% CO_2_ atmosphere. The colonies grown on overnight agar plate incubation were spotted onto a target slide and prepared according to the manufacturer's instructions for analysis using VITEK MS® system software version 3.0 (bioMérieux). This system was termed the standard identification method (sID). The colonies grown on overnight agar plates were also used for the preparation of the inoculum disk diffusion test (Kirby–Bauer disk diffusion susceptibility test protocol), according to CLSI [6]. This test was termed the standard disk diffusion method (sAST). The bacterial identification and antimicrobial resistance detection results obtained using this conventional workflow were used for comparison in the data analyses.

### 2.4. Rapid Modified Bacterial Identification (rID) from Positive Blood Culture

The rapid modified bacterial identification from positive blood cultures evaluated was previously described by Chen et al. [7]. In brief, 3 mL of positive blood culture broth was aspirated from positive blood culture bottles using a sterile syringe and transferred to a 10 mL serum separator tube. The aspirate was centrifuged for 5 min at 3,000 rpm, the supernatant was discarded, and 20 *μ*L of the bacterial pellet was transferred to the center of a chocolate agar plate (bioMérieux). The inoculum was streaked out to form a 2 by 2 cm, and the dish was incubated at 35°C in 5% CO_2_ atmosphere, for up to 4–6 h. Growth on the plate was recovered with a 1 *μ*L inoculating loop, to obtain a sufficient inoculum to be spotted onto a target slide prepared, according to the manufacturer's instructions for analyses using MALDI-TOF VITEK MS® system software version 3.0 (bioMérieux). The identification process was performed only once for each sample. Criteria for interpretation of the results proposed by the manufacturer were a single organism identification (same genus and species) as successful identification, and more than one species results with the same genus, as acceptable identification.

### 2.5. Rapid Modified Antimicrobial Susceptibility Testing (rAST) from Positive Blood Culture

A modification of the standard disk diffusion method (rAST) was evaluated for the detection of antimicrobial resistance. The rAST followed CLSI standards in all aspects, except for the inoculum preparation. CLSI guideline recommends preparing the inoculum for AST with a direct suspension of isolated colonies selected from 18–24 h agar plate incubation [6]. In this study, we used colonies selected from a rapid culture grown (4–6 h agar plate incubation). The inoculum was prepared from the rapid culture grown on a 150 mm Mueller–Hinton Agar (bioMérieux, France), and then, disks were applied and plates incubated at 35°C ± 1° and incubated for 18 h. The following antimicrobial agents were evaluated for GN: amikacin 30 *μ*g, amoxicillin-clavulanate 20/10 *μ*g, ampicillin 10 *μ*g, ampicillin-sulbactam 10/10 *μ*g, cefepime 30 *μ*g, ceftazidime 30 *μ*g, cefuroxime 30 *μ*g, ciprofloxacin 5 *μ*g, gentamicin 10 *μ*g, meropenem 10 *μ*g, piperacillin-tazobactam 100/10 *μ*g, and trimethoprim-sulfamethoxazole 23.75/1.25 *μ*g (Oxoid®, Thermo-Fisher, USA). For GP, cefoxitin 30 *μ*g, clarithromycin 15 *μ*g, clindamycin 2 *μ*g, doxycycline 30 *μ*g, erythromycin 15 *μ*g, gentamicin 10 *μ*g, levofloxacin 5 *μ*g, rifampicin 5 *μ*g, and trimethoprim-sulfamethoxazole 23.75/1.25 *μ*g (Oxoid®) were evaluated. The inhibition zones were analyzed after 18–24 h, and the results were interpreted by CLSI proposed breakpoints [6].

### 2.6. Data Analysis

Bacterial identification and antimicrobial resistance detection results obtained by the rapid modified methods were compared with those obtained by standard methods. Bacterial identification results were classified as correct identification at species or genus levels, and nonreliable identification and the success rate of rID were calculated. Sensitive (S), intermediate (I), and resistant (R) interpretative results were evaluated for each antimicrobial agent tested by disk diffusion methods. The categorical agreement (CA) between rAST and the current standard method was determined. Categorical discrepancies were classified as a very major error (VME) or a false-susceptible result; a major error (ME) or a false-resistant result; and a minor error (mE) when one method yielding an intermediate result and the other yielding a susceptible or resistant result. The acceptable intermethod categorical discrepancies rates of VME, ME, and mE are ≤1.5%, ≤3%, and ≤10%, respectively [8].

### 2.7. Statistical Analysis

The concordance of antimicrobial resistance results was determined using the categorical agreement and discrepancies rates for the detection of antimicrobial resistance with 95% confidence intervals (CI). The 95% CIs for the proportion of categorical agreement between the rAST and the sAST, including VME, ME, and mE, were also calculated. Kappa coefficients were calculated using a 95% CI. Kappa interpretation is as follows: <0, less than chance agreement; 0.01–0.20, slight agreement; 0.21–0.40, fair agreement; 0.41–0.60, moderate agreement; 0.61–0.80, substantial agreement; 0.81–0.99, almost perfect agreement. All statistical analyses were performed using SPSS Versions 20.0.

## 3. Results

In total, 524 bacterial isolates from blood cultures, 246 GN and 278 GP, were analyzed. The overall bacterial concordance rate by rID method for the genus level and species level was 88.6% and 87.3%, respectively (Table 1). The highest concordance rate was observed among GN isolates (96%). *K. pneumoniae* and *E. coli* presented of 96% and 99% of concordance, respectively. *P. aeruginosa* and *A. baumannii* complex showed a 100.0% and 93% of bacterial identification concordance, respectively. *S. aureus* presented 99%, and coagulase-negative Staphylococci (CNS) presented the lower rate of concordance in the rID method (78%) (Table 1).

A total of 2196 and 1476 antimicrobial agents determinations were obtained for GN and GP isolates, respectively. The overall CA, VME, ME, and mE were 97.7, 0.7, 0.5, and 1.1% for GN and 98.0, 0.5, 0.7, and 0.8% for GP, respectively (Table 2). When we analyzed these categorical discrepancies by antimicrobial agents used for GN, we observed that all antimicrobial agents presented acceptable results, but piperacillin-tazobactam VME was ≥1.5. Meropenem CA, VME, and ME were 98.3, 0.5, and 0.5%, respectively, and no mE was observed. When we analyzed these categorical discrepancies by antimicrobial agents used for GP, we observed that all antimicrobial agents presented acceptable limits, but the clarithromycin VME value was 1.8%, and levofloxacin ME value was 3.7%. Oxacillin CA, ME, and mE were 97.4, 1.2, and 0.6%, respectively, and VME was observed (Table 2).

When we analyzed these categorical discrepancies by bacterial species, we observed that all bacteria species presented results in accordance to acceptable limits. *Enterobacteriaceae* presented a CA, VME, ME, and mE of 97.4, 0.4, 0.3, and 1.4%, respectively. *K. pneumoniae* and *E. coli* presented a CA, VME, ME, and mE of 99.1, 0.4%, 0.2%, and 0.6% and 97.1, 0.3, 0.2, and 2.5%, respectively. *P. aeruginosa* presented a CA of 97.9, a VME of 1.2%, and no ME and mE detection. Staphylococci group presented a CA 98% *Staphylococcus aureus* and coagulase-negative Staphylococci (CNS) presented a CA, VME, ME, and mE of 99.1, 0.1, 0.3%, and 0% and 97.2, 0.2%, 0.5%, and 0.7%, respectively.

The Kappa scores of 2196 comparitive results of rapid and standard antimicrobial agent determinations for GN bacteria isolates (*n* = 183) ranged from 0.85 to 0.99 (Table 2). The Kappa scores of the results of antimicrobial agent determinations for *K. pneumoniae* (*n* = 73) isolates and *E. coli* (*n* = 54) isolates ranged form 0.91 to 0.97. The Kappa scores of 2196 comparative results of rapid and standard antimicrobial agent determinations for GN ranged from 0.87 to 0.99 (*p* < 0.001).

## 4. Discussion

Rapid identification and antimicrobial susceptibility testing for bacteria causing bloodstream infections are of utmost importance to reduce morbidity and mortality. Infectious diseases have a substantial global health impact. Bloodstream infection is still among the ten most common causes of deaths in developed countries [9]. Incidence rates between 80 and 257 per 100,000 person-year have been reported, with higher rates in the more recent years [10, 11], and the case fatality rate constitutes 10–40% [12, 13]. In spite of great treatment efforts during the last decades, infections are still among the most common causes of death worldwide. In the last decades, a rising occurrence of microorganisms resistant to antimicrobial agents is of particular concern [14].

Current technologies employed in routine diagnostics are based on bacterial culture, which constitutes the actual gold standard. Although those methods are precise and sensitive, they are rather slow. We modified previously developed bacterial identification and antimicrobial susceptibility testing methods from blood culture to propose a faster, easier, and reliable method to reduce the turnaround time of bloodstream infection diagnosis in a routine microbiology laboratory. The proposed method in this study was distinguished from standard ones by centrifugation and short-time grown on solid media.

The analyses of the correct identification at species or genus levels were calculated. The correct bacterial identification rate was higher among GN isolates. Rapid identification of nosocomial Enterobacteriaceae (*K. pneumoniae*) and nonfermentative Gram-negative bacteria (*P. aeruginosa*) from blood cultures is clinically relevant because it improves the management of bloodstream infections. The CNS presented the lower correct bacteria identification, mainly at the species level. The main reason for that could be explained by the fact that most of GP was slower growing than GN species, pointed by more GP isolates that did not grow during the 6 h of incubation. The clinical impact of CNS identification at the species level requires caution, considering the doubtful of this bacteria group as causing bloodstream infections or as a blood collection contaminant [15].

Multiple methods of direct identification from positive blood cultures have been proposed with correct identification to the species level ranging from 67% to 93% [16–21]. Commonly found with these published procedures is the ability to correctly identify Gram-negative bacteria more frequently than Gram-positive bacteria, a finding we also encountered. However, the methods that have been employed in literature are often laborious, typically requiring an extraction procedure with centrifugation, lysis, or filtration of the specimen. Advantages of our protocol include its simplicity and speed, requiring only 5 to 10 min for preparation of the smudge plate. The results are available within 4–6 h, and the procedure could be easily incorporated into the routine microbiology laboratory workflow.

In our study, interpretative results of disk diffusion methods were evaluated for each antimicrobial agent tested by rAST. We obtained lower rates of VME, ME, and mE for GN and GP. In addition, rAST presented acceptable values according to intermethods rates guidelines. When we analyzed these categorical discrepancies by antimicrobial agents used for GN, we observed that only piperacillin-tazobactam showed VME above acceptable limits. For GP, only clarithromycin and levofloxacin showed categorical discrepancies above acceptable limits. Overall, kappa scores of the results of the comparisons of rAST and sAST demonstrated an excellent concordance between the two methods.

In conclusion, the bacteria identification concordance rate, CA, kappa coefficient, and the accuracy of rAST were accepted to be adopted in the routine since the intermethod error rates were above those acceptable [8]. The rapidity and reliability were factors in its adoption for routine use, allowing us to save up to 24 h in identifying bacteria from blood culture and supplying useful information to adapt antibiotic therapy when necessary. Faster diagnosis of pathogens is critical to guarantee adequate therapy for infections. Delay in the initiation of appropriate antibiotic therapy has been recognized as a risk factor for mortality. Ferrer et al. evidenced a significant association between delay in antibiotic administration over the first 6 hours after the identification of patients with severe sepsis and septic shock and increasing mortality. These results underscore the importance of early identification and treatment of septic patients in the hospital setting. As mentioned often in the literature, sepsis is a time-dependent condition and should be recognized as an urgent situation that requires an immediate response [22]. Empirical treatment with broad-spectrum antibiotics is started in patients suspected of bacteremia. However, diagnostics are necessary, as recent studies have observed that 25 to 33% of patients are inappropriately treated within the first 24 h due to lack of coverage from organism resistant to broad-spectrum antibiotics. Improved patient care includes rapid bacterial identification and appropriate susceptibility results for blood cultures [23]. In addition, the rapid and accurate antimicrobial susceptibility test is paramount to the management of patients with severe infections. The ability to report identification and susceptibility results from positive blood cultures shortly after they signaled positive for growth is of great value in reducing time to appropriate therapy [4, 24]. Otherwise, the current culture-based AST tools rely on time-consuming culturing techniques, followed by disk diffusion and broth dilution susceptibility testing. In many clinical microbiology laboratories, agar disk diffusion is routinely used, while automated AST instruments are limited in the number of antibiotics, concentrations tested, and lack the capability of analyzing polymicrobial samples or heterogeneous response of bacterial populations to the antibiotics [25]. Meanwhile, agar disk diffusion tests present the same technical difficulties, as they cannot figure out the heterogeneous response of bacterial populations and polymicrobial analysis. Novel methods have been described to counteract those technical difficulties [26, 27].

In light of ever-increasing problems related to the emergence of multidrug-resistant bacteria, rapid microbiological diagnostics are of growing importance. Timely pathogen detection and availability of susceptibility data are essential for optimal treatment. Identification and susceptibility by the rapid phenotypic method showed a high degree of accuracy. The marked reduction in time to results may have significant implications for patient care.

## Figures and Tables

**Table 1:** Identification performance of rapid bacterial identification (rID) versus the standard bacterial identification method (sID).

Organism ID by the current standard method	*N* (%) identified in Vitek MS (rID)			
	Concordance level			
	*n*	Species	Genus	No. Id
Overall	524	459 (87.3)	466 (88.6)	58 (11)
Gram-negative bacteria	246	235 (96)	235 (96)	11 (4)
*K. pneumoniae*	100	96 (96)	96 (96)	4 (4)
*E. coli*	67	66 (99)	66 (99)	1 (4)
*K. oxytoca*	15	13 (87)	13 (87)	2 (13)
*E. cloacae*	6	6 (100)	6 (100)	0
*P. mirabilis*	5	5 (100)	5 (100)	0
*E. hormaechei*	4	4 (100)	4 (100)	0
*S. marcescens*	3	3 (100)	3 (100)	0
*C. koseri*	2	1 (50)	1 (50)	1 (50)
*M. morganii*	1	1 (100)	1 (100)	0
*P. aeruginosa*	22	22 (100)	22 (100)	0
*A. baumannii*	14	13 (93)	13 (93)	1 (7)
*B. cepacia*	4	3 (75)	3 (75)	1 (25)
*S. maltophilia*	3	3 (100)	3 (100)	0
Gram-positive bacteria				
Staphylococci	278	224 (81)	231 (83)	47 (17)
*S. aureus*	73	72 (99)	72 (99)	1 (1)
*Coagulase-negative staphylococci*	205	153 (75)	160 (78)	45 (22)

Concordance rate for species level and genus level and nonreliable identification by Vitek MS system.

**Table 2:** Antimicrobial susceptibility testing performance of rapid antimicrobial susceptibility testing (rAST) compared with the standard method (sAST).

Antimicrobial agents	CA (%)	VME (%)	ME (%)	mE (%)	Kappa score
*GN*					
Amikacin	98.3	1 (0.5)	1 (0.5)	0 (0)	0.96
Amoxicillin-clavulanate	98.1	0 (0)	1 (0.5)	2 (1.1)	0.95
Ampicillin	97.0	0 (0)	1 (0.5)	4 (2.2)	0.85
Ampicillin-sulbactam	97.8	0 (0)	1 (0.5)	8 (4.4)	0.93
Cefepime	96.9	2 (1.1)	1 (0.5)	1 (0.5)	0.97
Ceftazidime	97.2	1 (0.5)	1 (0.5)	2 (1.1)	0.96
Cefuroxime	97.2	1 (0.5)	1 (0.5)	2 (1.1)	0.93
Ciprofloxacin	97.8	1 (0.5)	1 (0.5)	1 (0.5)	0.94
Gentamicin	97.5	2 (1.1)	1 (0.5)	0 (0)	0.95
Meropenem	98.3	1 (0.5)	1 (0.5)	0 (0)	0.97
Piperacillin-tazobactam	93.0	4 (2.2)	1 (0.5)	5 (2.7)	0.90
Trimethoprim-sulfamethoxazole	98.3	2 (1.1)	0 (0)	0 (0)	0.99
Total	97.7	15 (0.7)	10 (0.5)	25 (1.1)	

*GP*					
Clarithromycin	95.5	3 (1.8)	1 (0.6)	1 (0.6)	0.91
Erythromycin	99.0	0 (0)	1 (0.6)	0 (0)	0.99
Clindamycin	98.0	2 (1.2)	0 (0)	0 (0)	0.87
Doxycycline	100	0 (0)	0 (0)	0 (0)	0.91
Rifampicin	100	0 (0)	0 (0)	0 (0)	0.99
Gentamicin	96.6	1 (0.6)	0 (0)	4 (2.4)	0.99
Levofloxacin	91.9	1 (0.6)	6 (3.7)	2 (91.2)	0.91
Oxacillin	97.4	0 (0)	2 (1.2)	1 (0.6)	0.95
Trimethoprim-sulfamethoxazole	95.6	0 (0)	2 (1.2)	4 (2.4)	0.91
Total	98	7 (0.5)	11 (0.7)	12 (0.8)	

Categorical agreement (CA), very major error (VME), major error (ME), and minor error (mE) per antibiotic agents used for Gram negative (GN) and Gram positive (GP). Kappa scores of the 2196 antimicrobial agent determinations result of rAST for Gram-negative bacteria isolates and 1476 antimicrobial agent determinations result of rAST for Gram-positive bacteria isolates.

## Data Availability

The data used to support the findings of this study are available from the corresponding author upon request.
